# Management of Chronic Heart Failure in Dialysis Patients: A Challenging but Rewarding Path

**DOI:** 10.31083/j.rcm2506232

**Published:** 2024-06-25

**Authors:** Luxuan Guo, Yue Ji, Tianhao Sun, Yang Liu, Chen Jiang, Guanran Wang, Haitao Xing, Bo Yang, Ao Xu, Xian Xian, Hongtao Yang

**Affiliations:** ^1^First Teaching Hospital of Tianjin University of Traditional Chinese Medicine, 300193 Tianjin, China; ^2^National Clinical Research Center for Chinese Medicine Acupuncture and Moxibustion, 300193 Tianjin, China; ^3^Tianjin University of Traditional Chinese Medicine, 301617 Tianjin, China; ^4^Dongzhimen Hospital, Beijing University of Traditional Chinese Medicine, Institute of Nephrology & Beijing Key Laboratory, 100700 Beijing, China

**Keywords:** dialysis, heart failure, risk factors, drug management

## Abstract

Chronic heart failure (CHF) is a common complication and cause of death in dialysis patients. Although several clinical guidelines and expert consensus on heart failure (HF) in the general population have been issued in China and abroad, due to abnormal renal function or even no residual renal function (RRF) in dialysis patients, the high number of chronic complications, as well as the specificity, variability, and limitations of hemodialysis (HD) and peritoneal dialysis (PD) treatments, there are significant differences between dialysis patients and the general population in terms of the treatment and management of HF. The current studies are not relevant to all dialysis-combined HF populations, and there is an urgent need for high-quality studies on managing HF in dialysis patients to guide and standardize treatment. After reviewing the existing guidelines and literature, we focused on the staging and diagnosis of HF, management of risk factors, pharmacotherapy, and dialysis treatment in patients on dialysis. Based on evidence-based medicine and clinical trial data, this report reflects new perspectives and future trends in the diagnosis and treatment of HF in dialysis patients, which will further enhance the clinicians’ understanding of HF in dialysis patients.

## 1. Introduction

Heart failure (HF) and end-stage renal disease (ESRD) frequently coexist [1, 2]. Approximately half of the patients with HF have concomitant chronic kidney disease (CKD) [3]. Up to 70% of patients with CKD and 36% of patients with ESRD requiring dialysis have HF [4]. Studies have estimated [5] that the incidence of HF is 12–36 times higher in dialysis patients compared to the general population. Approximately half of all deaths in dialysis patients are due to cardiovascular (CV) disease, but data regarding the management of HF in end-stage kidney disease (ESKD) patients receiving dialysis remains limited [6, 7]. The American Kidney Foundation’s Quality of Renal Outcomes Initiative identified left ventricular (LV) systolic dysfunction and left ventricular hypertrophy (LVH) as independent predictors of poor survival in dialysis patients and recommended that sustained normovolemia should be the cornerstone of HF management in dialysis patients [8]. The 2021 European Society of Cardiology guidelines provide Class IIA recommendations for ESKD and refractory volume-overloaded patients to use renal replacement therapy as an option for HF treatment [9]. The inferior cardiac and renal function or even absence of residual renal function (RRF) and the high number of complications in dialysis patients, together with the specificity, variability, and limitations of hemodialysis (HD) and peritoneal dialysis (PD), make HF in dialysis patients very different from that in the general population in terms of treatment and management of risk factors. Most studies have excluded dialysis patients with ESRD due to safety and tolerability considerations. Against this background, this report searched databases and relevant guidelines to summarize the staging and diagnosis of HF in dialysis patients, the management of risk factors, medications, and dialysis treatments, and to review the challenges and opportunities in managing HF in dialysis patients. This report will help to enhance the clinicians’ understanding of HF in dialysis patients and to standardize the clinical management of HF in dialysis patients.

## 2. Diagnosis and Classification of HF in Dialysis Patients

Commonly used diagnostic methods for dialysis-combined HF [5] include symptoms and physical examination, X-ray, electrocardiogram, echocardiogram, biomarkers, cardiac magnetic resonance, cardiac computed tomographic (CT)/computed tomographic angiography (CTA), and laboratory tests. Questionnaires [10] and biomarkers of cardiac inflammatory fibrosis [11] are novel predictive methods of HF in patients with CKD. In the future, it will still be necessary to search for more specific prognostic indicators in dialysis patients and combine them with routine examinations to make therapeutic management decisions.

HF is a group of complex clinical syndromes characterized by abnormal changes in the structure and function of the heart due to various causes, resulting in ventricular dysfunction, which leads to a group of complex clinical syndromes manifested by fatigue, weakness, dyspnea, and fluid retention (pulmonary circulation congestion, somatic circulation congestion, and peripheral edema) [5, 9]. Dialysis is a life-sustaining treatment for patients with ESRD and severe acute kidney injury (AKI). Volume overload and dyspnea during dialysis cannot be attributed to HF alone, and their severity varies with renal replacement therapy/ultrafiltration (RRT/UF). Therefore, there are limitations in applying New York Heart Association grading criteria to these patients [12]. To remedy this problem, the Acute Dialysis Quality Initiative working group [13] proposed HF grading for dialysis patients that considers the assessment of HF symptoms and dialysis cycles in dialysis patients. This grading system suggests that the degree of HF in a patient be graded by the evaluation of dyspnea before and after RRT/UF, with the grading scheme representing the usual level of dyspnea in the patient. If the grading is the same before and after RRT/UF, it is recommended that the assessment be performed after treatment, as shown in Table 1.

**Table 1.** **ADQI task force recommendations for grading heart failure in dialysis patients**.

Grade	Grading criteria
Level 1	Echocardiography confirms heart disease without symptoms
Level 2R	Exertional dyspnea may be relieved to NYHA class I by RRT/UF
Level 2NR	Exertional dyspnea not relieved by RRT/UF to NYHA Class I
Level 3R	Dyspnea from activities of daily living relieved by RRT/UF to NYHA Class ${\mathrm{II}}^{a}$
Level 3NR	Dyspnea from activities of daily living not relieved by RRT/UF to NYHA Class II
Level 4R	Dyspnea at rest may be relieved by RRT/UF to NYHA class ${\mathrm{III}}^{a\,b}$
Level 4NR	Dyspnea at rest not relieved by RRT/UF to NYHA class III

^a^ If dyspnea symptoms improve to NYHA class I level, the patient will be classified as class 2R; ^b^ If dyspnea improves to NYHA class II level, the patient will be classified as class 3R. ADQI, Acute Dialysis Quality Initiative; NYHA, New York Heart Association; RRT/UF, renal replacement therapy/ultrafiltration.

## 3. Risk Factor Management of HF in Dialysis Patients

### 3.1 Volume Overload

**Fig. 1.**
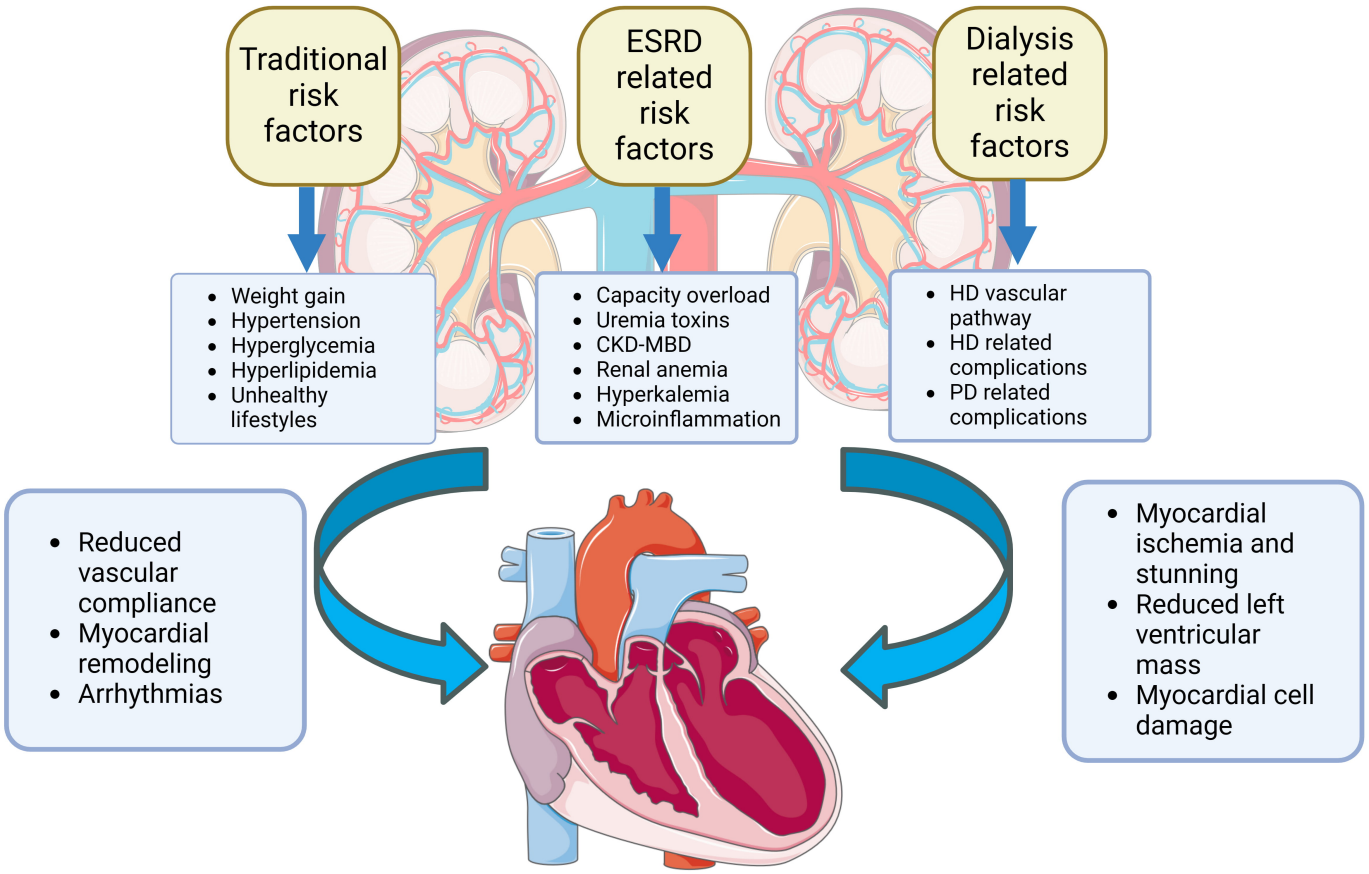
**Risk factors for HF in dialysis patients include those that are similar to those in the general population, as well as those related to ESRD and dialysis.** Early identification of these risk factors is important in preventing HF in dialysis patients. CKD-MBD, chronic kidney disease-mineral and bone disorder; HF, heart failure; ESRD, end-stage renal disease; HD, hemodialysis; PD, peritoneal dialysis.

Fig. 1 shows the causes of heart failure in dialysis patients. Hypervolemia is common in dialysis patients due to the high fluctuation of sodium and water during and between dialysis treatments. Volume overload is considered to be the most common complication in ESRD patients and is directly associated with multiple complications, including interdialytic weight gain and blood pressure during the interdialytic period, atherosclerosis, LVH, increased cardiac afterload, congestive heart failure, pulmonary congestion, and a persistent inflammatory oxidative stress state [14, 15, 16]. Volume overload is a major cause of HF and death in dialysis patients [17], and HF due to volume overload is the leading cause of re-hospitalization in patients on continuous ambulatory peritoneal dialysis (CAPD) [18]. The dry weight of dialysis patients occurs when the maximum fluid reduction can be achieved through dialysis ultrafiltration without hypotension. The criteria for dry weight includes (1) no obvious hypotension during dialysis; (2) effective control of blood pressure before dialysis; (3) no clinical edema; (4) no signs of pulmonary congestion on chest X-ray; (5) cardiothoracic ratio: male <50%, female <53%. Conditional HD centers can also apply bioelectrical resistance methods to assess whether the patient’s dry weight meets these standards. Chest X-ray, brain natriuretic peptide (BNP), N-terminal pro-brain natriuretic peptide (NT-proBNP), lung ultrasound, extracellular fluid assessment, and bioelectrical impedance analysis are commonly used to assess the dry weight of dialysis patients. Lung ultrasound is more suitable for patients with excessive blood volume; bio-impedance is more widely used in clinical practice [19]. Various bio-impedance measurement devices may have variability in terms of accuracy and reproducibility when detecting HD patient volume [20, 21]. The reliability of bioelectrical impedance in detecting volume overload in dialysis patients remains to be further investigated [22, 23, 24]. Extracellular fluid is highly accurate in the assessment of dry weight, but the cost for this methodology is high and is mainly used for scientific research [25]. The most accurate measurement method has yet to be defined, and the results of the studies are still controversial [26, 27, 28]. Volume overload is a significant risk factor for HF in dialysis patients. During volume overload, ventricular end-diastolic volume increases, and ventricular myofibers are stretched, which can be compensated by the Frank-Starling mechanism in the early stage. But, myocardial dysfunction will occur with chronic high-volume loading, ultimately altering ventricular remodeling [29, 30]. Volume assessment consists of the patient’s history and physical examination, with physical examination being the primary method of volume assessment. The history should carefully ask the patient about symptoms associated with hypotension on dialysis, blood pressure control during the interdialytic period, and symptoms related to hypervolemia. The physical examination should include the patient’s edema, dyspnea, degree of jugular venous filling, lung auscultation, weight, and blood pressure. These examinations should be reviewed and performed at least once a month, with the optimal frequency individualized by the patient’s condition [25]. Regularly monitoring volume status and body composition in children is essential to ensure that target weights are adjusted to match growth [31]. After restricting salt intake according to the patients’ urine output, physical activity, body weight, and nutritional status (as recommended in the 2021 Kidney Disease: Improving Global Outcomes (KDIGO) guidelines [32], for CKD patients, salt intake should be <5 g/day), if the patient’s weight continues to increase during the treatment interval, consideration should be given to strengthening the dialysis regimen. For specific dialysis regimens, please refer to the dialysis management section of this report. Wearable artificial intelligence devices for measuring volume status and heart rhythm, are currently being developed for use in clinical practice [33].

### 3.2 Abnormal Blood Potassium

A cohort study showed that hypokalemia before and after HD was associated with increased mortality in dialysis patients [34]. Studies have shown that significant changes in blood potassium levels before and after HD increase the risk of death in these patients. Therefore, it is important to increase the frequency of blood potassium monitoring and to individually adjust fluctuations in blood potassium concentration in the dialysate [35]. Patients undergoing dialysis are at increased risk of developing hyperkalemia, which is often caused by poor dietary compliance (high potassium diets) or inadequate intestinal potassium excretion, reduced urine output, inadequate dialysis, transfer of potassium ions from intracellular to extracellular compartments, use of multiple medications, especially renin-angiotensin system inhibitors (RASi) and loop diuretics, and comorbidities such as diabetes mellitus (DM) and ESRD which can lead to elevated blood potassium levels due to metabolic acidosis [36, 37]. The lower risk of hyperkalemia in PD patients compared to HD patients is due to better continuity of treatment in PD patients [38]. Patients treated with PD retained RRF longer and use higher diuretic doses than HD patients [39]. Consequently, PD patients are at higher risk for hypokalemia [40]. Studies have shown that reducing the $K^{+}$ concentration in the dialysate can be used in HF patients with high blood potassium levels before dialysis. It should be noted that the significant difference in $K^{+}$ concentration between serum and the dialysate may exacerbate rapid changes in blood potassium, resulting in an increased risk of cardiac conduction instability and malignant arrhythmias in HD patients [41]. Future research is needed to investigate how the use of a dialysate with a lower $K^{+}$ concentration can continuously improve the state of hyperkalemia in patients with pre-dialysis hyperkalemia. In addition, new potassium binders such as high-sodium cyclic silicate, patiromer, and sodium zirconium cyclosilicate powder (ZS)-9, which act faster and improve $K^{+}$ excretion, are worth promoting in treating hyperkalemia in HF patients undergoing HD [42, 43].

### 3.3 Renal Anemia

Anemia is an independent risk factor for all-cause mortality and CV events in HD and PD patients [44, 45, 46]. Renal anemia can lead to the progression of LVH and is significantly correlated with left ventricular mass (LVM) in dialysis patients [47]. The leading causes of renal anemia include deficiency of endogenous human erythropoietin (EPO), iron deficiency, microinflammatory conditions, secondary hyperparathyroidism, inadequate dialysis, and other causes of bleeding and anemia [48]. The treatment for renal anemia often involves improving the nutritional status, using erythropoiesis stimulants (ESAs), iron preparations, hypoxia-inducible factor prolyl hydroxylase inhibitors (HIF-PHI), and levocarnitine. High-dose ESAs can increase the risk of CV events, death, and tumor recurrence [49, 50]. According to the Chinese guidelines for renal anemia, ESAs are not recommended for patients with a hemoglobin (Hb) level above 90 g/dL who also have HF and CKD [47]. The 2022 American College of Cardiology/American Heart Association/Heart Failure Society of America (ACC/AHA/HFSA) guidelines recommend that ESAs are not recommended for the treatment of anemia in patients with HF [12]. HIF-PHI is a newly developed small molecule oral drug for the treatment of renal anemia. It can stimulate the production of EPO within physiological levels, while simultaneously down regulating the levels of Serum hepcidin. This promotes the intestinal absorption, transport, and utilization of iron, reducing the dosage of iron. It is applicable to dialysis patients who respond poorly to ESA therapy [51] and has the potential to become an oral alternative to traditional ESA therapy [52]. A multicenter, prospective, randomized controlled trial in dialysis patients in China has shown that both Roxadustat and epoetin alfa can effectively raise Hb levels in patients with HD and PD [53]. Studies suggest that the difference between the two is that Roxadustat does not require a dose increase when used in HD patients with microinflammation [54]. However, there is a lack of evidence regarding the targets of HIF-PHI in treating renal anemia and iron supplementation. Further research is needed to fill this gap. L-carnitine is widely used to treat anemia in dialysis patients, improving anemia and microinflammatory status, reducing the need for ESAs [55, 56], and improving cardiac function and LVH in HD patients [57]. In addition, there are new drugs such as Ziltivekimab, which can significantly improve inflammatory markers, increase serum albumin levels in HD patients, reduce the need for ESAs, and improve therapeutic hypo-responsiveness to ESAs [58].

### 3.4 Calcium and Phosphorus Metabolism Disorders

Disorders of calcium and phosphorus metabolism often coexist with secondary hyperparathyroidism. As kidney function declines, the activity of vitamin D in the kidneys also diminishes. The deficiency of active vitamin D affects the intestinal absorption of calcium, leading to hypocalcemia [59], which stimulates the parathyroid glands to secrete parathyroid hormone [60]. In maintenance hemodialysis (MHD) patients, secondary hyperparathyroidism is prevalent, with an incidence of over 50%. Elevated levels of parathyroid hormone are associated with an increased risk of death from hypertension and cardiovascular events [61]. Insufficient dialysis or reduced glomerular filtration rate (eGFR) can lead to hyperphosphatemia. This condition can cause bone metabolic disorders, stimulate the secretion of parathyroid hormone by the parathyroid gland, and induce myocardial fibrosis [62]. Studies have shown that hyperphosphatemia in dialysis patients is an independent risk factor for vascular calcification [63]. Chronic hyperphosphatemia can stimulate the transformation of vascular smooth muscle cells into bone-like cells, leading to the calcification of the media in arteries. Decreased oxygen supply to the arteries and myocardial fibrosis can lead to rupture or occlusion, affecting the blood supply and oxygen delivery to the heart. This is a common complication among chronic HD patients and is also one of the key factors increasing the risk of CV events and mortality in HD patients [64, 65].

Currently, numerous methods exist to decrease vascular calcification in dialysis patients, such as the utilization of vitamin K1 [66], subcutaneous insulin and heparin [67], as well as the use of sodium thiosulfate [68], bisphosphonate [69], and inositol hexaphosphate hexasodium salt (SNF) 472 [70]. These drugs have been tested for treating vascular calcification in dialysis patients and have proven to be effective. However, most of these drugs are used in HD patients, and additional studies on vascular calcification are still needed to determine the appropriate dose and frequency in PD patients. Three measures are needed to prevent and treat elevated blood phosphate in dialysis patients. The first is to reduce the intake of phosphorus-rich foods such as dairy products, meat, nuts, and hidden food additives. An open-ended, multi-center interventional clinical study randomly divided MHD patients into two groups: a strict control of blood phosphate group (where blood phosphate levels were controlled at 3.5–4.5 mg/dL) and a standard control group (where blood phosphate levels were maintained at 5.0–6.0 mg/dL). After 12 months compared to the standard control group, the strict control group of blood phosphate had significantly reduced coronary artery calcification (CAC) scores, suggesting that more stringent control of blood phosphorus in MHD patients may potentially delay the progression of CAC [71]. The second method is the use of phosphate binders, which can be calcium-containing or non-calcium-containing phosphate binders. Due to the risk of promoting vascular calcification associated with calcium-containing phosphate binders, non-calcium-containing phosphate binders such as lanthanum carbonate, sevelamer, vascular calcification, Iron (II) hydroxide, and ferric citrate are currently used in clinical practice [72, 73, 74]. However, these drugs may cause adverse gastrointestinal reactions. Newer types of drugs, such as EOS789 and tenapanor, are being developed for use in dialysis patients [75, 76, 77]. Third, adequate dialysis can increase the removal of phosphorus by increasing the dialysis dose and prolonging the dialysis time, which can reduce blood phosphorus [78]. There is still considerable controversy about treating hypocalcemia in dialysis patients, and there is insufficient evidence to prove the efficacy of calcium mimetics. Individualized treatment methods should be adopted to treat hypocalcemia in dialysis patients, and patients with significant symptomatic hypocalcemia could still benefit from correction to prevent adverse complications [79]. The treatment of hypercalcemia in dialysis patients includes the discontinuation of calcium, calcitriol, active vitamin D, and the use of calcium-free or low-calcium dialysis fluid [80, 81]. The 2017 KDIGO guideline [82] recommends that for CKD G5D patients, a dialysate calcium concentration of 1.25–1.50 mmol/L is recommended.

### 3.5 Micro-Inflammation and Oxidative Stress

Inflammation is believed to be the primary mechanism underlying CV events in patients with renal insufficiency [83, 84, 85]. A micro-inflammatory state refers to the process where toxins stimulate the production of various inflammatory factors, which persist in the blood, causing mild inflammation. This micro-inflammatory state is persistent low-level inflammation characterized by elevated levels of inflammatory factors [86]. Uremic toxins can directly stimulate the increase of superoxide dismutase (SOD) and reactive oxygen species (ROS), enhance lipid peroxidation, and exacerbate oxidative stress [87]. Studies [88, 89, 90] have found that inflammatory and oxidative stress factors such as high-sensitivity C-reactive protein (hs-CRP), interleukin 6 (IL-6), tumor necrosis factor-alpha (TNF-α), SOD, midbrain dopamine (MDA), and glutathione peroxidase (GSH-Px) are closely related to vascular calcification, atherosclerosis, CV events, anemia, and death in HF patients undergoing dialysis. Studies [91] have shown that early intervention in a micro-inflammatory state can decrease CV complications in HD patients and alleviate anemia and malnutrition. *In vitro* studies [92, 93] have shown that the uremic toxin indolyl sulfate (IS) and p-cresol (PC) serum can induce vascular endothelial cell dysfunction. In addition, animal experiments have found that [94] increased ROS caused by mitochondrial respiratory impairment is an essential mechanism of ventricular dysfunction caused by excessive volume overload. Studies [95] have found that increased oxidative stress due to the activation of nicotinamide adenine dinucleotide phosphate (NADPH) oxidase and the activation of the RhoA/Rho kinase (ROCK) pathway were closely related to the increased risk of CV events in dialysis patients. There are currently various methods to improve the inflammatory and oxidative stress state of dialysis patients, such as the oral medications N-acetylcysteine [96], indoloazepine [97], and lanthanum carbonate [98], the use of biocompatible peritoneal dialysis fluid [99, 100, 101], the use of hydrogen-rich dialysis fluid with a pH value close to physiological levels and low levels of glucose degradation products (GDP) and advanced glycation end products (AGEs), the use of icodextrin peritoneal dialysis solution, the use of Hemo Filtration with endogenous Reinfusion (HFR) for hemodialytic filtration [102], and the use of vitamin E-coated dialysis membranes [103]. Increasing the frequency of dialysis and avoiding infection pathways (such as prophylactic use of antibiotics before and after PD catheterization [104]) can decrease inflammation and oxidative stress in dialysis patients. Additional clinical research on decreasing inflammation and oxidative stress will be needed, in addition to novel markers predicting the level of inflammation and oxidative stress in dialysis patients.

### 3.6 Traditional Risk Factors

Hypertension, constipation, advanced age, smoking, alcoholism, obesity, DM, and hyperlipidemia are traditional risk factors for CV events. The frequency of sympathetic nerve discharges in dialysis patients can be up to 2.5 times higher than in healthy subjects, and volume overload of more than 6% of body weight can lead to sympathetic activation [105]. According to the 2017 European Renal Association-European Dialysis and Transplant Association (ERA-EDTA)/European Society of Hypertension (ESH) consensus guidelines [106], dialysis patients with ambulatory blood pressure monitored for more than 24 h (not necessarily up to 44 h) and a mean blood pressure ≥130/80 mmHg is considered hypertensive. HD patients with a mean morning and evening home blood pressure ≥135/85 mmHg on six consecutive non-dialysis days are considered to be hypertensive. High-quality studies on the criteria for hypertension in dialysis patients are still scarce, and more individualized criteria for hypertension in dialysis patients should be developed in the future, taking into account age, pre- and post-dialysis blood pressure changes, and comorbidities. Compared with peri-dialysis BP and home BP monitoring, 44 h ambulatory blood pressure (BP) monitoring is the gold standard for BP measurement in HD patients [107], which can assess the patient’s BP rhythm during the inter-dialysis period and predict the risk of target organ damage and CV events [108]. Studies have shown that home BP monitoring provides similar results to ambulatory BP monitoring for inter-dialysis BP assessment [109] and is superior to routine in-center BP monitoring in predicting adverse outcomes [110]. Therefore, home BP monitoring may provide a more accurate assessment of BP in dialysis patients. An observational study [111] showed that lower systolic blood pressure (SBP) before dialysis and higher SBP during dialysis were associated with reduced mortality. In contrast, higher SBP before and higher SBP during dialysis were associated with increased mortality, although it is unknown whether there is a causal relationship between the two. Management of hypertension in dialysis patients should begin with volume management, adjustment of dialysate sodium concentration, and increased dialysis duration [112]. If the blood pressure remains uncontrolled, antihypertensive medication is required. Specific hypertension treatment protocols can be found in the Medication Management section of this report. Future studies detailing HF patients on dialysis should be undertaken to assess the relationship between BP and clinical outcomes and to determine the paradoxical U-shaped relationship between BP and mortality [113]. Additional studies are needed to determine the ideal BP range for HF patients on dialysis.

Dialysis patients are often at risk for coronary atherosclerosis. Elevated cholesterol levels in PD patients are independently associated with all-cause and cardiovascular disease (CVD) mortality [114]. Despite the high CV disease burden and lipid metabolism disorders that characterize patients with advanced kidney disease, treatment with statins has produced conflicting results in CV outcomes. The therapeutic effect of statins on CV disease in dialysis patients is still controversial, and several large-scale clinical trials have found that statins do not reduce the occurrence of CV events in dialysis patients [115, 116]. Recent guidelines [117, 118, 119, 120] do not recommend the use of lipid-lowering agents in dialysis patients but recommend patients who receive statin therapy at the beginning of dialysis continue to use statin therapy [121]. In addition, there is evidence [122, 123] that statins can increase vascular calcification, which is an important risk factor for HF in dialysis patients. Large-scale, high-quality clinical studies looking at the role of new lipid-lowering agents in dialysis patients are needed to address these issues. In addition, maintaining a healthy lifestyle, such as a healthy diet, regular exercise, smoking cessation, maintaining a healthy weight, and blood glucose levels are important preventive measures for HF in dialysis patients.

## 4. Drug Treatment for Dialysis Patients

The special characteristics of dialysis patients make them very different from the general population in terms of the treatment of HF and the management of their risk factors. In most randomised controlled trials (RCTs), the exclusion of dialysis patients due to clinician concerns about potential adverse drug reactions has resulted in insufficient evidence to support the use of medications in dialysis patients [124] (see Table 2 for details). In clinical practice, guideline drugs for HF in dialysis patients are usually reduced or not used because of safety, intolerability, and pharmacokinetic issues [125]. In the available randomised controlled trial (RCT) studies, there is a lack of large-scale, multi-center, prospective, high-quality clinical trials on the use of individual drugs or drug combinations for the treatment of HF in dialysis patients.

**Table 2.** **Common drugs for HF in dialysis patients**.

Drugs	Possible benefits	Potential risks
ACEI	Preservation of RRF, protection of peritoneum, improvement of left ventricular mass, reduction of urinary protein	Hyperkalemia, hypotension, increased blood creatinine
ARB	Preservation of RRF, protection of the peritoneum, improvement of left ventricular mass, reduction of urinary protein	Hyperkalemia, hypotension, increased blood creatinine
ARNI	Improvement in myocardial remodeling, reduction in myocardial markers, delayed decline in renal function	Hyperkalemia, hypotension
Beta-blocker	Improved LVEF and NYHA classification, improved myocardial remodeling	Hypotension, bradycardia
Spironolactone	Improved LVEF, improved left ventricular mass, improved myocardial remodeling	Hyperkalemia, decreased eGFR
Loop diuretic	Improved capacity loading without compromising RRF	Ototoxicity
Digoxin	Improved LV function and reduces heart rate	Arrhythmia
	Diuresis	

ACEI, angiotensin converting enzyme inhibitor; ARB, angiotensin II receptor blocker; HF, heart failure; RRF, residual renal function; ARNI, angiotensin receptor enkephalinase inhibitor; LVEF, left ventricular ejection fraction; NYHA, New York Heart Association; eGFR, reduced glomerular filtration rate; LV, left ventricular.

### 4.1 ACEI/ARB

There are two classes of RASi: angiotensin converting enzyme inhibitors (ACEIs) and angiotensin II receptor blockers (ARBs). RASi analogues can prevent the conversion of angiotensin I (Ang I) to Ang II, attenuate ventricular remodeling, reverse LVH and improve cardiac function, and have been shown to reduce the incidence of the composite outcome of hospitalization and all-cause death in HF and in the general population [126]. RASi analogues are also commonly used to treat early CKD to slow the progression of CKD and reduce the incidence of CV events [127]. However, RASi analogues have been excluded from most trials in patients with ESRD because of the risk of causing significant deterioration in renal function, hyperkalemia and hypotension [128]. Direct evidence of benefit from their use in patients with combined HF and dialysis is lacking, and the results of the available studies appear to be conflicting. RASi has been shown to reduce all-cause mortality by 11% in patients with HD [129]. A meta-analysis by Yang Y *et al*. [130] showed that ACEIs/ARBs were more effective in reducing left ventricular mass (LVMI) in HD but did not significantly improve left ventricular ejection fraction (LVEF) compared with controls. A large observational cohort study including 4879 patients with PD by Shen JI *et al*. [131] demonstrated that the use of an ACEI/ARB was associated with a reduced risk of all-cause mortality and composite endpoints including all-cause mortality, ischemic stroke, and myocardial infarction (MI). The FOSIDIAL trial included 397 patients with HD with LVH, and although cardiovascular events trended downward compared to the placebo group, fosinopril did not show a significant benefit in the composite cardiovascular event endpoint, possibly because patients in the fosinopril group had worse baseline comorbidities compared to the placebo group [132]. Chang *et al*. [133] showed that ACEIs were not only ineffective in reducing all-cause and cardiovascular mortality in MHD patients, but were associated with a higher risk of HF hospitalization. In none of the above studies were specific outcomes for patients with known HF mentioned. A retrospective cohort study of 4771 patients with long-term HD combined with HF showed that ACEI/ARB use was associated with lower all-cause and cardiovascular mortality [134]. The Italian multicenter randomized double-blind RCT conducted by Cice *et al*. [135] enrolling 332 patients with HD combined with heart failure with reduced ejection fraction (HFrEF) showed that the combination of telmisartan with standard ACEI/Beta-Blocker (BB)-based therapy significantly reduced all-cause mortality, cardiovascular mortality, and length of hospital stay in HF in chronic heart failure. In conclusion, RASi has potential benefits for HF treatment in dialysis patients; however additional RCTs are needed to guide its use in the dialysis population. In clinical practice, due to the side effects of RASi analogues, serum potassium concentration, renal function and blood pressure levels should be closely monitored when these drugs are used in dialysis patients.

### 4.2 Angiotensin Receptor–Neprilysin Inhibition

Sacubitril-valsartan (SV), the world’s first angiotensin receptor enkephalinase inhibitor (ARNI), is a sodium salt complex of sacubitril, an enkephalinase (NEP) inhibitor, and valsartan, an angiotensin II type 1 receptor blocker [136], which inhibits both the Renin-Angiotensin-Aldosterone System (RAAS) and NEP and has synergistic vasodilator, and antihypertensive effects. It has been shown to reverse cardiac hypertrophy, improve cardiac remodeling and promote water and sodium excretion [137]. Another advantage of ARNI is that it binds to plasma proteins and is not rapidly cleared by HD [138]. Most of the existing studies on the use of ARNI in patients with combined HF and dialysis have small cohorts, are non-randomized, retrospective, and lack high-quality RCTs. The subgroup analysis of the Prospective Comparison of ARNI with ACEI to Determine Impact on Global Mortality and morbidity in Heart Failure (PARADIGM-HF) trial, which included 8399 patients with HFrEF randomized to either SV or epitomize, showed that ARNIs reduced the risk of cardiac and renal events and death and slowed the rate of decline in eGFR better than ACEIs in patients with HF-combined CKD; however, the trial excluded patients with eGFR <30 mL/min/1.73 $m^{2}$ [139]. Another study in 21 patients with PD combined with heart failure with preserved ejection fraction (HFpEF) confirmed SV’s role in alleviating HF symptoms and reducing NT-proBNP. It showed a trend towards improvement in diastolic function, although echocardiographic parameters did not change significantly [140]. A Korean [141] retrospective study of 23 patients with HD and PD combined with HFrEF showed that SV improved LVEF and myocardial marker levels, however, the study was retrospective, had a small number of patients, and lacked a control group and clinical endpoints such as cardiovascular mortality. In contrast, a retrospective study of 247 patients with MHD combined with HFpEF showed that SV treatment significantly improved NYHA functional class in patients with MHD combined with HFpEF, both in terms of HF symptoms and levels of NT-proBNP and troponin I (TNI), and echocardiographic findings, with significant reductions in left atrial diameter (LAD), left ventricular posterior wall thickness in diastole (LVPWTd), left ventricular end-diastolic diameter (LVEDD), left ventricular end-diastolic volume (LVEDV) and left ventricular end-systolic volume (LVESV), suggesting that SV can reverse LV remodeling [142]. The results of a retrospective study that included 64 patients with PD and 38 patients with HD showed that although there was no significant difference in echocardiographic parameters from pre and post controls after initiation of SV therapy, LVEF was significantly improved in the SV group compared to the control group, and subgroup analyses showed that both myocardial markers and LVEF improved significantly in PD patients compared to HD patients, but this study was not conducted in patients with known HF [143]. Niu *et al*. [144] evaluated 31 patients with HD and 18 patients with PD combined with HFrEF. The results in this case-control study showed that SV improves left ventricular systolic and diastolic function in patients with dialysis-combined HF, and that there were no differences between SV and conventional therapy in terms of adverse events, such as hyperkalemia and hypotension, and in terms of rates of hospitalization for cardiovascular disease or other causes.

However, in stark contrast to the above studies suggesting that ARNIs are beneficial for patients with combined HF on dialysis, a subgroup analysis of a retrospective multicenter study of 618 dialysis patients with HFrEF showed that the use of ARNI increased the risk of hospitalization for HF (HR, 1.97 [1.36–2.85]) as well as the combined risk of hospitalization for HF and all-cause mortality, compared to an ACEI/ARB (HR, 1.73 [1.23–2.44]) [145]. The shortcomings of this study include the failure to consider both inpatients and outpatients, its retrospective nature, and the short duration of follow-up. ARNI may be potentially beneficial for patients with HF on dialysis, and the ongoing phase 4 multicenter randomized open-label trial “The Sacubitril/Valsartan for CKD 5-stage dialysis patients with heart failure” aims to compare whether blood pressure is superior to irbesartan in patients randomized to SV dialysis, as well as survival, cardiac function, renal function, and adverse effects. “The effect of Sacubitril/Valsartan on cardiovascular events in dialysis patients and efficacy reduction of baseline LVEF value” will assess the role of SV versus RASi for cardiovascular events in patients with HD and PD combined with HF.

### 4.3 Beta-Blocker

There are many types of beta-blockers, including those that are poorly dialyzed (atenolol, bisoprolol, vinblastine) moderately dialyzed (carvedilol, labetalol, and propranolol), highly cardio-selective (atenolol, bisoprolol, and metoprolol) and less cardio-selective (propranolol, carvedilol, labetalol). A meta-analysis [146] studied 75,193 PD and HD patients using dialysis-compatible beta-blockers (DBBs) and non-dialysis-compatible beta-blockers (NDBBs), and the use of DBBs and NDBBs on the risk of all-cause mortality, major adverse cardiac events (MACE), acute myocardial infarction (AMI), and HF in dialysis patients. The results showed that the use of DBBs and NDBBs had no effect on the risk of all-cause mortality, total MACE, and AMI in dialysis patients, and compared with NDBBs, DBBs were associated with a significant reduction in the risk of HF. Another meta-analysis [147] also showed that DBBs and NBBs had similar mortality rates, but DBBs reduced the risk of CV events. However, some studies reached the opposite conclusion. A propensity-matched retrospective cohort study comparing dialysis clearance and morbidity and mortality in dialysis patients on different medications showed that in HD patients, the use of DBBs is associated with an increased risk of death in the subsequent 6 months compared with NDBBs [148]. It has been suggested [149] that the use of cardio-selective BB may be associated with fewer CV events and lower all-cause mortality compared with dialysis patients on non-selective BB. A propensity-matched retrospective cohort study [150] of 3400 HD patients with HF showed lower all-cause mortality in patients treated with BB and even lower mortality in patients treated concomitantly with BB and ACEIs or ARBs. A meta-analysis [151] evaluated the effects of BB on CV events and mortality in dialysis patients and found that BB significantly reduced the incidence of CV events and mortality in dialysis patients. However, some studies [152, 153] suggested that different types of BB had less efficacy in dialysis patients. BB have many applications in CKD and chronic heart failure (CHF). However, the treatment options for dialysis patients are still limited, and the benefits and potential risks of BB for dialysis patients are still uncertain. Therefore, the current use of BB in clinical practice is still based on patient tolerability and availability.

### 4.4 Digoxin

The use of digoxin in patients with ESRD remains controversial. It is important to note that digoxin has a narrow therapeutic window. The therapeutic dose is close to the toxic dose, and digoxin is mainly excreted by the kidneys, with a higher likelihood of toxicity when used in dialysis patients. Therefore, its use in dialysis patients requires routine monitoring of digoxin concentrations, with adjustment or assessment of the need to continue the patient’s use of digoxin in light of RRFs and the pattern of renal replacement therapy [154]. A retrospective study of 120,864 HD patients [155] showed that digoxin was associated with increased mortality, with a significantly increased risk of death if the pre-dialysis blood potassium concentration was <4.3 mmol/L and the digoxin concentration was ≥1 µg/L. Continuous hemodialysis may be an option in ESRD patients with digoxin toxicity [156]. Based on the above studies, we do not recommend the routine use of digoxin, but only when AF is not effectively controlled in patients with HF. The clinical evidence for digoxin in patients with HF combined with dialysis remains limited, and further studies are needed on the prevention and treatment of digoxin toxicity and its adverse effects in dialysis patients.

### 4.5 Diuretics

Spironolactone is both a diuretic and a salicorticoid receptor antagonist, which inhibits the activation of the RAAS system in patients with HF and has hypotensive, diuretic, and potassium-elevating effects, and can be used in dialysis patients with RRF [157, 158]. According to several small clinical trials, the addition of low-dose spironolactone (25 mg/d) to the treatment regimen of most HD patients is safe and effective in reversing LVH, improving CV function and potassium fluctuations, and reducing the risk of CV and all-cause mortality [159, 160]. In 16 patients with HD with HFrEF, the use of spironolactone (25 mg /day) significantly improved cardiac function, reduced left ventricular mass and cardiovascular mortality, and did not increase hyperkalemia [161]. Unfortunately, the randomized, double-blind, placebo-controlled pilot study by Charytan *et al*. [160] that included 129 HD patients failed to find a cardiovascular benefit for spironolactone in HD patients, even though only 21 patients in this study had a diagnosis of congestive heart failure at baseline. A meta-analysis [162, 163] found that aldosterone antagonists may decrease the risk of all-cause mortality and CV mortality in ESRD patients requiring HD or PD, and may also reduce the incidence of CV and cerebrovascular diseases without a significant risk of hyperkalemia. In PD patients, due to hypokalemia, using a 25–75 mg/d aldosterone antagonist therapy can effectively elevate blood potassium levels, reduce the need for oral potassium, and decrease systolic blood pressure. It also inhibits the damage caused by bacterial peritonitis and prevents vascular calcification [164]. There have been a few RCTs conducted on patients with PD combined with HF. The results of a small RCT on CAPD with HFrEF (n = 18) showed that spironolactone significantly improved mean LVEF without increasing the risk of hyperkalemia [165]. However, as most of the current studies are small-sample, single-center studies, whether 25 mg/d is the ideal dose in terms of safety and efficacy remains unknown. Data from more prospective, large-scale, multicenter clinical trials are needed to determine the optimal dose and confirm clinical efficacy. We believe two large, multicenter clinical trials (NCT03020303, NCT01848639) will provide more compelling data.

Loop diuretics are frequently used for hypertension and volume management in patients with CKD and HF and may help increase urine output and electrolyte excretion in dialysis patients with some residual urine output. Loop diuretics act on the thick segment of the ascending branch of medullary collaterals and inhibit the Na-K-2CL cotransporter, inhibiting NaCl reabsorption and acting as a diuretic [166]. In dialysis patients, loop diuretics are reduced compared to non-dialysis patients. The dose of loop diuretics remains uncertain due to impaired hepatic and renal function, which leads to a prolonged half-life of loop diuretics, drug-drug interactions, reduction of organic anion-transporting proteins, and ototoxicity in high-dose loop diuretics [167]. A retrospective clinical study [168] found that compared to patients who did not continue to use loop diuretics after starting dialysis, patients who continued to use loop diuretics during the first year of dialysis had a lower hospitalization rate, a lower incidence of dialysis-related hypotension (IDH), and a lower interdialytic weight loss compared to the control group. However, there was no significant difference in the mortality rate during the first year of dialysis. A meta-analysis [169] examined the effect of loop diuretics on IDH in maintenance dialysis patients and found that loop diuretics reduced the incidence of IDH, all-cause mortality, and CV mortality. Although loop diuretics are commonly used diuretics to improve volume overload in dialysis patients, the efficacy of loop diuretics is poor in anuric renal disease patients, and ototoxicity is common. High-quality studies involving loop diuretics are needed to verify their clinical efficacy and safety in dialysis patients [170].

Tolvaptan, a diuretic that selectively antagonizes arginine pressing V2 receptors and increases free water excretion by inhibiting water reabsorption in the collecting ducts, effectively reduces intra- and extracellular fluids, with the significant advantage of being less likely to cause deterioration in renal function [171]. Several studies have shown that treatment with tolvaptan prolongs the time to initiation of dialysis in CKD stage 4–5 patients with comorbid HF [172, 173]. The use of tolvaptan in dialysis patients with RRF has been found to increase urine output with a favorable safety profile [171, 174]. However, it should be noted that the use of this drug in patients with ESRD is still relatively small, the dosage and efficacy of the drug are still uncertain, and relevant studies are needed to better determine their clinical application.

### 4.6 Sodium-Dependent Glucose Transporters 2 Inhibitor (SGLT2i)

Two multicenter, randomized, double-blind, placebo-controlled RCTs-the Dapagliflozin and Prevention of Adverse Outcomes in Heart Failure (DAPA-HF) study and the Empagliflozin Outcome Trial in Patients with Chronic Heart Failure and a Reduced Ejection Fraction (EMPEROR-Reduced) trial-have demonstrated that empagliflozin reduces cardiovascular event mortality and HF hospitalization in patients with HFrEF, regardless of the presence of diabetes mellitus [175, 176]. The results of the EMPEROR-Preserved Trial demonstrated that engeletin similarly reduced cardiovascular event mortality and HF hospitalization in patients with HFpEF, and reduced the risk and severity of HF events [177]. Unfortunately, the DAPA-HF study excluded patients with eGFR <30 mL/min/1.73 $m^{2}$, and the EMPEROR-Reduced trial and EMPEROR-Preserved Trial excluded patients with eGFR <20 mL/min/1.73 $m^{2}$ and dialysis. There is a lack of evidence for the use of SGLT2i in dialysis patients, and it is hoped that the ongoing evidence for the Safety of Dapagliflozin in Patients with Hemodialysis-Combined Heart Failure (SDHF) trial (NCT05141552) and the DAPA-HD trial (NCT05179668) will provide relevant data on the use of dapagliflozin in HD patients.

## 5. Heart and Kidney Transplantation

Patients on dialysis with severe HF are at high risk for complications after undergoing heart transplantation. Shoji *et al*. [178] showed that the risk of all-cause mortality was five times higher in patients undergoing dialysis after heart transplantation. Post-transplant dialysis makes these patients more susceptible to complications, and therefore, concomitant heart and kidney transplantation (SHKT) is often recommended for patients with end-stage HF complicated by dialysis.

SHKT is used in patients with severe HF and advanced renal insufficiency. A clinical study comparing older (≥60 years, n = 53) versus younger (<60 years, n = 47) recipients, as well as recipients on preoperative dialysis (n = 49) and those not on dialysis (n = 51), showed that SHKT was safe in patients aged 60 years and older or younger, with or without dialysis dependence [179]. Schaffer *et al*. [180] performed a retrospective analysis of the United Network for Organ Sharing (UNOS) database and showed that patients with end-stage HF combined with dialysis had a higher post-transplant survival rate with SHKT than patients with a preference for matched heart transplant alone (HTA).

## 6. Left Ventricular Assist Devices (LVADs)

LVAD implantation is usually not recommended in dialysis-dependent ESKD patients because of concerns about poor patient prognosis and increased mortality due to complications associated with LVAD implantation. Kirklin *et al*. [181] showed that renal dysfunction before LVAD implantation was associated with higher mortality rates after implantation, and that survival rates progressively decreased with higher degrees of renal insufficiency. In patients with severe renal dysfunction and patients with severe renal dysfunction and other major comorbidities, the use of a temporary device for initial support while awaiting organ recovery before implantation of a long-term circulatory support device may be considered. An 11-year study conducted by Bansal *et al*. [182] showed that 81.9% of patients with ESRD before LVAD implantation died during the follow-up period (compared with 36.4% of patients without ESRD), with a median time to death of 16 days after implantation (2125 days in patients without ESRD). Lower pulsatile blood pressure in patients with continuous-flow LVAD implants may lead to ventricular arrhythmias due to low ventricular volumes and low pressures during dialysis, with a higher risk in patients with HD compared to those with PD [183]. Although current evidence suggests that dialysis HF patients undergoing LVAD implantation have a poorer prognosis and lower survival, the application of PD or intermittent HD may be a more prudent option in this high-risk population.

## 7. Implantable Cardioverter Defibrillators (ICD)

There is a lack of RCTs on the use of ICDs in patients with HD on dialysis, and only a few observational studies have evaluated the efficacy of ICDs in patients with combined HFrEF on dialysis. In a matched cohort study including 303 dialysis patients, the application of ICDs in dialysis HFrEF patients did not result in a significant survival benefit [184]. In contrast, the results of a study including 100 dialysis patients with LV dysfunction showed a significant reduction in all-cause mortality with the use of an ICD compared with patients without an ICD (HR, 0.40 [0.19–0.82]). A subgroup analysis of patients with an LVEF <35% (n = 91) similarly demonstrated that the use of an ICD significantly reduced the risk of all-cause mortality (HR, 0.32 [0.15–0.71]) [185].

Prophylactic use of ICDs did not reduce sudden cardiac death or all-cause mortality in dialysis patients without significant left ventricular ejection fraction (LVEF >35%), Greater than 50% of patients died during follow-up, with the main causes of death being infection and sudden cardiac death [186]. Subcutaneous ICD implantation may be a safer alternative and has been found to reduce the risk of infection associated with transvenous ICD implantation in dialysis patients, but there was no significant difference in all-cause mortality or length of hospital stay [187]. Observation data shows that compared to ICD users without ESKD, ESKD patients who receive dialysis simultaneously with ICD have a significant increase in overall mortality and incidence of complications [188]. Further exploration of strategies to reduce complications in ESRD patients undergoing ICD implantation is needed.

## 8. Cardiac Resynchronization Therapy (CRT)

Due to the lack of relevant RCTs and conflicting findings on the role of CRT on patients with dialysis combined with HF, the effectiveness of CRT in these patients remains unknown, and more evidence specific to patients with dialysis combined with HF is needed. A case-control study evaluating the efficacy and safety of CRT in 14 patients with HD and 1 patient with PD combined with HFrEF demonstrated that CRT increased all-cause mortality and all-cause hospitalization rates but did not significantly affect the rate of HF hospitalization compared with controls [189]. However, a large retrospective study of nearly 11,000 patients with HFrEF combined with advanced CKD (stages 3–5), including dialysis patients, showed a significant reduction in the risk of death with the use of CRT combined with a defibrillator [190].

## 9. Dialysis Treatments

### 9.1 Differences between HD and PD Regimens

Unlike HD, PD removes excess fluid and sodium from the body continuously and slowly, with less impact on hemodynamics and avoids the risk of HF associated with vascular access [191]. In the case of right HF (RHF), using a peritoneal dialysis catheter as an access point to drain ascites allows better control of ascites, facilitates reduction of intra-abdominal pressure, and results in better protection of cardiac and renal function [192]. A retrospective clinical study [193] found a significant increase in eGFR and a decrease in systolic blood pressure in PD-treated CHF patients compared to HD patients, but PD patients had a significantly increased risk of CV death and no difference in overall survival. A meta-analysis [194] of 28 trials showed a significant short-term CV benefit in HD patients compared with PD patients, reducing the risk of hypertensive HF, CHF, myocardial tonicity, and atrial fibrillation, but there was no difference in overall survival. Clinical trials [195, 196] have shown no difference in overall BP control and survival in HD patients compared with PD patients. There is insufficient clinical evidence to confirm the difference between PD and HD in the control of HF in dialysis patients. Factors currently influencing decision-making include patient preferences for lifestyle and participation in the dialysis process and advice from the nephrologist.

### 9.2 HD Management Program for Dialysis Combined with HF

#### 9.2.1 Arteriovenous Fistula Management

Arteriovenous fistula (AVF) is the preferred vascular access for CHF compared to an arteriovenous graft (AVG) and a central venous catheter. However, after performing an AVF, local hemodynamic changes may occur, and some of the blood flow enters the vein directly through the AVF pathway rather than through the capillary bed, leading to inadequate effective cardiac output and “Arteriovenous fistula steal syndrome” induced HF [197]. AVFs and AVGs are prone to stenosis of the access vessels, which can lead to graft dysfunction, inadequate dialysis, and access thrombosis [198]. High-flow AVF can cause HF, and clinical manifestations of HF, such as chest tightness, dyspnea, nausea, and vomiting, may occur when there is impaired myocardial contractile function associated with high-flow AVF [199, 200]. AVF/AVG formation is associated with significant right atrial dilatation and remodeling and an increased risk of HF episodes and death [201]. The 2019 edition of the Kidney Disease Outcomes Quality Initiative (KDOQI) Clinical Practice Guideline for Vascular Access strongly recommends routine clinical monitoring of AVF and AVG (e.g., clinical signs, physical examination, dialysis adequacy) to detect clinical signs of vascular access dysfunction [30]. Hypervolemic HF is one of the complications after AVF. Maintenance of the AVF may require ligation to reduce vascular access. Other techniques include placing blood clips on the venous supply, blood reflux reduction maneuvers, or Miller’s procedure [202]. The 2019 Chinese Expert Consensus on HD Vascular Access [203] recommends that pre-procedural HF assessment be performed in all patients with established vascular access, that patients at high risk of HF receive regular follow-up, and that AVF/AVG placement is not recommended for dialysis patients with a preoperative ejection fraction (EF) <30%. To prevent vascular access-related HF, AVF/AVG should be avoided in patients at a high risk of HF or with pre-existing HF, and forearm AVF/AVG placement should be preferred whenever possible. The use of end-to-side anastomosis and end-to-end anastomosis may be beneficial to avoid a side-to-side anastomosis and results in higher blood flow and higher graft patency [204]. However, the 2019 European Renal Best Practice (ERBP) guidelines [205] concluded that there is not yet sufficient evidence to prove the superiority of an end-to-end anastomosis over a side-to-side anastomosis. In patients with an established AVF/AVG, we believe timely monitoring of AVF/AVG flow and echocardiography should be performed in the event of HF manifestations or worsening of pre-existing HF symptoms. New vascular access devices facilitate the continuation of HD in patients with vascular system failure, increase vascular access patency [206, 207], and improve dialysis access-related complications.

#### 9.2.2 HD Mode Optimization

**Fig. 2.**
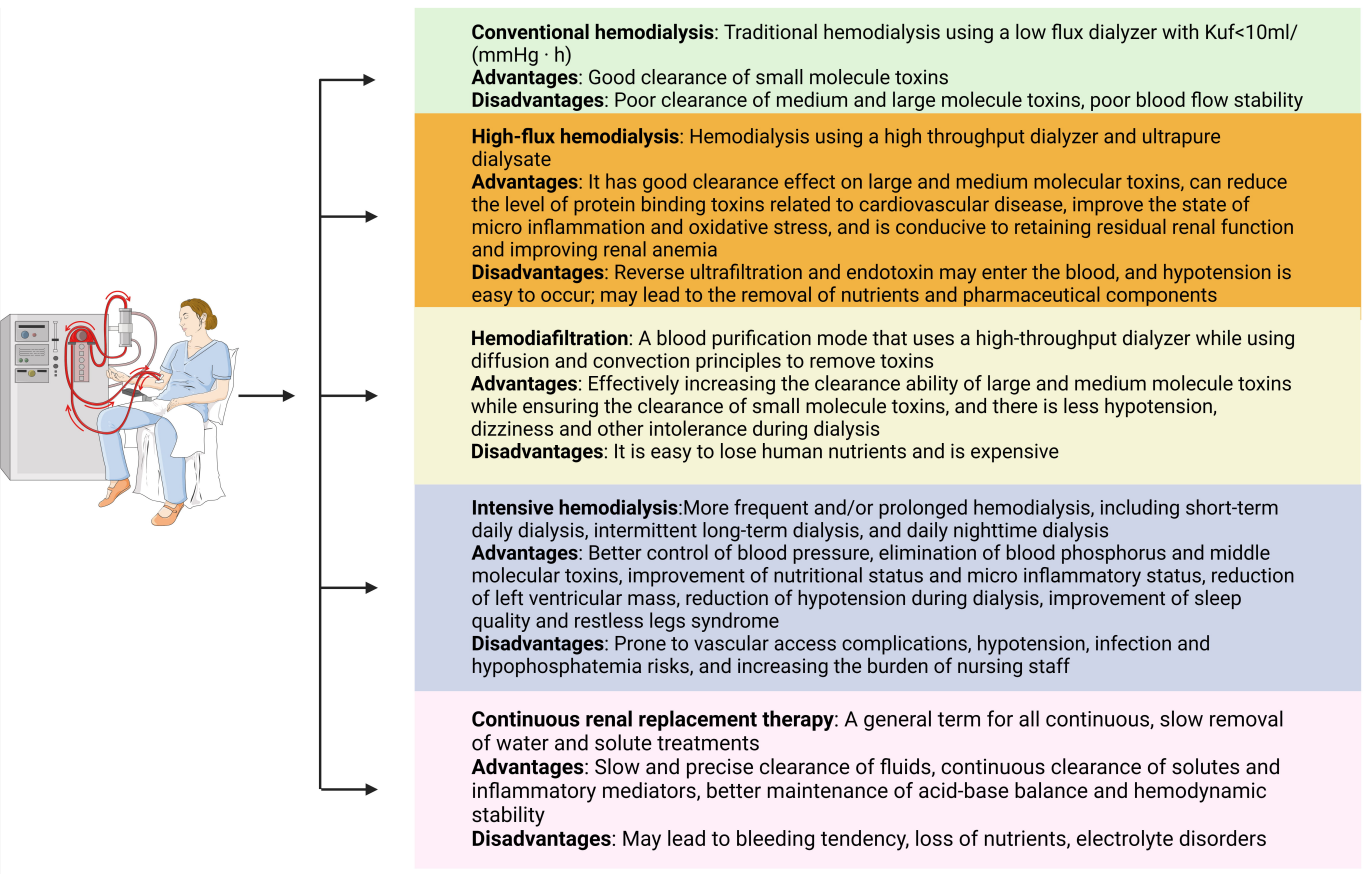
**The advantages and disadvantages of different HD modes.** HD, hemodialysis.

HD-related complications include volume overload, myocardial ischemia and myocardial dysfunction, manifested by elevated troponin T (TnT), IDH, cardiac diastolic dysfunction, hemodynamic abnormalities and ultimately progression to myocardial injury, arrhythmia or sudden cardiac death, which are strongly associated with the risk of mortality [208]. The accumulation of sodium and water in patients with HD contributes to volume overload and hypertension, which is a significant risk factor for increased LVH and mortality [209]. There are multiple ways to optimize the mode of HD (Fig. 2). A clinical study [210] found that the volume overload status of dialysis patients improved significantly when the dialysis modality was changed from conventional HD to short-duration hemodialysis (SDHD). It has been shown [211, 212] that in dialysis patients who experience a sudden onset of swelling and uremic symptoms when the duration and frequency of dialysis are reduced, an appropriate increase in the frequency and duration of dialysis to intensify HD may result in more adequate dialysis. This may reduce the risk of IDH, hyperkalemia, hyperphosphatemia, anemia and HF, but how this is achieved and whether increasing the frequency or duration of dialysis is of more significant clinical benefit to the patient remains uncertain. A meta-analysis [213] showed that the introduction of nocturnal dialysis improves LVH, reduces the use of antihypertensive drugs and improves quality of life compared with CHD. Clinical studies [214, 215] have shown that convective therapy, use of cold dialysate (usually 34.0 °C–35.5 °C) and low sodium dialysate (usually <138 mEq/L), reduction of body weight during the inter-dialytic period, glucose infusion during HD, and use of midodrine can maintain hemodynamic stability, improve IDH and tissue perfusion, and reduce the incidence of myocardial ischemia during dialysis, but studies are still controversial [216].

Studies have shown [217, 218] that high-flux HD has many advantages over low-flux HD, including biocompatibility of dialysis membranes, better preservation of renal function, reduced inflammatory oxidative stress, and more efficient removal of macromolecular and intermediate-molecular uremic toxins, which may improve symptoms such as hypertension, anemia, pruritus and calcium-phosphorus metabolism disorders and reduce mortality. However, the current study is still controversial [219], and more studies with larger sample sizes, higher quality and extended follow-up are needed. Hemodialysis filtration (HDF) is an advanced dialysis technique that achieves a combination of diffusion and convection. This modality is more conducive to maintaining hemodynamic stability, improves cardiac remodeling, and directly reduces the circulation of relatively small and medium to large molecule uremic toxins, which reduces the risk of mortality [220]. Nevertheless, some studies have found no significant difference between this modality and HD [221, 222]. Clinical trials are underway (ISRCTN10997319, NTR7138 NTR) to determine whether high-volume dilute HDF reduces mortality compared with high-flux HD, and an answer is expected shortly. The use of on-line hemodiafiltration (OL-HDF) overcomes the technical challenges of bagged replacement dialysate and reduces costs [223]. Compared with high-flux HD, it can more effectively remove uremic toxins with a broader range of molecular weights [224] and reduce all-cause and CV mortality, and is associated with a better clinical prognosis [225]. However, some studies have concluded that this dialysis modality is not significantly different from other modalities [226]. The effects of different dilution modes, dilution ratios and flow rates [227] during dialysis with OL-HDF on solute clearance [228, 229] and dialysate quality [230] in HF in dialysis patients requires further investigation. Home HD [231] is a potential therapeutic option with potential benefits in terms of improved LVH, stabilization of blood pressure, increased rates of urinary toxin excretion, improved patient quality of life and reduced medication burden for the patient, which may be associated with a slower ultrafiltration rate and increased frequency of dialysis. However, the challenges of this modality of dialysis are the increased cost of caregiver time and training and the lack of research data on HF in dialysis patients.

### 9.3 Optimization of PD Programs

Clinical studies suggest that volume overload is an independent predictor of CV events, all-cause mortality and CV death in dialysis patients [17]. Improved ultrafiltration can improve the problem of volume overload in dialysis patients. The specific measures include increasing the concentration of glucose in the dialysate, using the icodextrin peritoneal dialysis solution, reducing the time the dialysate is stored in the abdomen, increasing the dialysis dose, adjusting the treatment modalities of peritoneal dialysis and combining PD with HD therapy. Three main concentrations of glucose dialysate are used in PD patients: 1.5%, 2.5% and 4.25%. Increasing the glucose concentration raises osmolality and improves ultrafiltration capacity, but 2.5% and 4.25% are more likely to damage the peritoneum, causing peritoneal inflammation and fibrosis. The 4.25% concentration of dialysate is mainly used in patients with urgent and sudden volume overload [232]. The Icodextrin peritoneal dialysis solution is more effective in improving the biocompatibility of the dialysate without sodium sieving and is superior to hypertonic dextrose solution in improving ultrafiltration. Its effect of increasing ultrafiltration is more pronounced in patients with high peritoneal solute transport and higher mean transport [99], and in patients with PD who have difficulty maintaining an average volume due to inadequate peritoneal ultrafiltration. Once-daily icodextrin peritoneal dialysis solution to prolong abdominal storage time is effective in improving volume overload [233]. Auguste and Bargman [233] found that in patients with poor RRF and inadequate dialysis, more water and solute removal can be achieved by increasing the total single dialysate dose, increasing the number of dialysis sessions, and reducing the abdominal storage time per bag. Unfortunately, increasing the total volume of dialysate increases glucose uptake, increasing the risk of hyperglycemia, hyperlipidemia and the risk of peritoneal sclerosis. Shortening the time each bag of dialysate is stored in the abdomen may result in inadequate solute removal. Increasing the dialysate dose is divided into incremental and maximal dose methods. Several studies have concluded that incremental dialysis is protective against RRF and helps to reduce the risk of peritonitis and improve patients’ quality of life [234, 235]. However, both treatments remain controversial in terms of inadequate solute clearance and volume overload, patient survival and peritonitis [236].

**Fig. 3.**
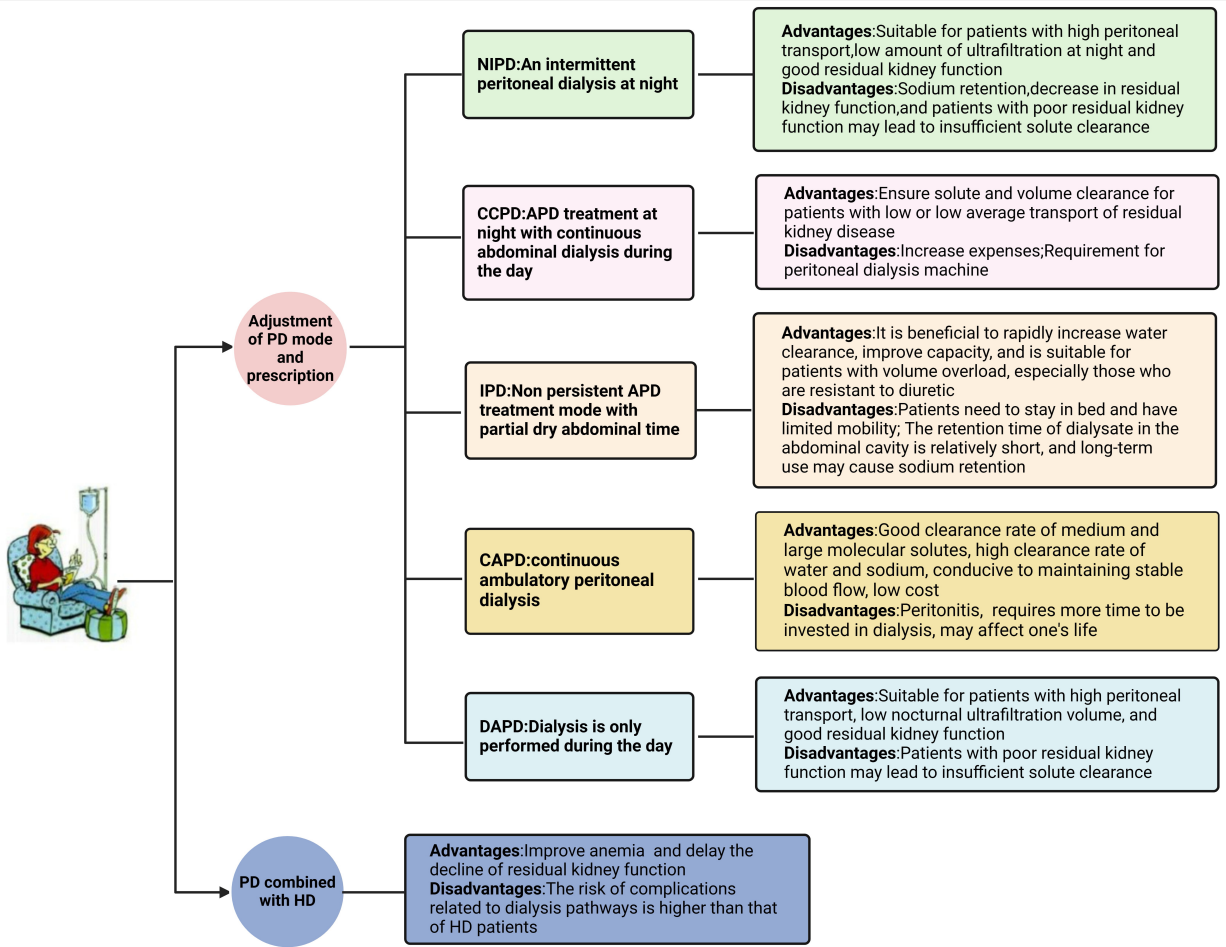
**The advantages and disadvantages of different PD modes.** HD, hemodialysis; PD, peritoneal dialysis; NIPD, nocturnal intermittent peritoneal dialysis; CCPD, circulation PD mode; APD, automated peritoneal dialysis; CAPD, continuous ambulatory peritoneal dialysis; DAPD, ambulatory peritoneal dialysis; IPD, intermittent peritoneal dialysis.

Depending on whether they rely on machine operation, all PD that rely on peritoneal dialysis machines for operation are collectively referred to as automated peritoneal dialysis (APD). The corresponding treatments are manual peritoneal dialysis, such as intermittent peritoneal dialysis (IPD), CAPD, and ambulatory peritoneal dialysis (DAPD). APD consists of multiple modes, such as continuous circulation PD mode (CCPD), IPD, and tidal peritoneal dialysis (TPD) (Fig. 3). If required, we can also combine APD with manual peritoneal dialysis. Because APD machines are expensive and vary according to the economic level of a country and government health policy [237], their use varies considerably between countries and regions. Different dialysis modalities have other effects on both solute and water removal [238]. An increasing number of studies have concluded that APD has the following advantages over CAPD: lower mortality, increased technical survival, improved peritonitis and quality of life [239, 240, 241]. However, there is still much controversy regarding survival, solute and volume removal and RRF protection [242, 243].

The Chinese HF guidelines for dialysis patients suggest that when PD patients have volume overload, the peritoneal transport function of PD patients can be assessed based on the peritoneal balance test by excluding the patient’s rapid deterioration of cardiac function, peritonitis, catheter mechanical factors, and excessive water and sodium intake, and that for patients with low or low average transport, continuous circulation peritoneal dialysis CCPD or PD combined with HD can be used. For patients with high transit or high average transit, HD is required if ultrafiltration fails, and APD, CAPD, DAPD, or manual IPD can be used if it does not fail. In patients with PD with HF with severe volume overload, especially those who are diuretic-resistant, have high transit function and reduced nocturnal ultrafiltration, an IPD can be used for a short period if the RRF is better, which is favorable for a rapid increase in water clearance. However, this mode results in a large amount of wasted time due to frequent exchange and short retention of dialysate in the peritoneal cavity. In addition, with prolonged use, there is often more water than sodium clearance due to the temporary retention of dialysate in the peritoneal cavity [244]. Patients with high peritoneal transit, low nocturnal ultrafiltration, and good RRF can be treated with DAPD or nocturnal intermittent peritoneal dialysis (NIPD). Nevertheless, NIPD may compromise renal perfusion due to rapid ultrafiltration and hemodynamic instability. In this case, CCPD can be used to ensure solute and volume clearance and avoid the sodium sieve effect, but the cost is much higher. It is not suitable for long-term use [245]. Multicenter, randomized clinical trials [246, 247] have shown that timely, complete, full-cycle management of patients using the remote monitoring capabilities of automated remote PD management (RPM-APD) reduces the rate of associated complications, with better patient compliance and potentially lower rates of technical failure compared with APD treatment. However, dialysis outcomes are equivalent between the two modalities. A cross-sectional clinical study found better water intake and blood pressure control in RPM-APD patients than in ESRD patients on CAPD [248]. Additional long-term and large-scale studies are needed to determine the effectiveness of RPM-APD.

If the patient’s dialysis is still inadequate, we recommend that the physician should consider other factors such as the cumulative volume effect of the patient’s comorbidities such as DM, HF, and vascular disease, and suggest the patient transition to HD if persistent volume overload continues after PD mode adjustment. Finally, for reasons such as the inability of pure PD and HD treatment to achieve satisfactory efficacy, to reduce the occurrence of dialysis complications, or to transition to HD treatment, combined HD and PD therapy may be adopted. A retrospective clinical study conducted in Japan [249] found that combination therapy was associated with lower all-cause mortality, CV mortality, and CHF-related mortality rates. Compared to patients with pure PD, these patients were able to transition to HD more rapidly, potentially due to the improved ability to manage volume overload. Patients receiving PD combined with HD treatment were at a lower risk of complications related to dialysis access compared to those receiving only PD treatment [250]. There was no significant difference in hospitalization risk, CV events, and congestive HF mortality between PD combined with HD treatment and HD [251], but PD combined with HD treatment had a higher risk of dialysis access-related complications than HD. These controversial findings support additional high-quality research to verify this risk.

## 10. Challenges and Future Research Directions

At present, most of the studies on heart failure in dialysis patients have been performed on hemodialysis patients. Heart failure and end-stage renal disease always interact with each other. One disease tends to aggravate the other disease, but modern research often separates dialysis patients and heart failure patients, resulting in insufficient evidence for relevant clinical research. At present, many clinicians regard dialysis as a transitional therapy for transplantation. Dialysis merely delays the disease and symptom deterioration rather than further improving the patient’s condition. Moreover, there is a large gap in medicine in different regions. One of the main reasons for the lack of evidence for HF treatment in dialysis patients is the lack of high-quality clinical RCT data on HF in dialysis patients. In addition, some studies excluded high-risk dialysis patients from clinical RCT studies that administered HF drugs and equipment interventions, making it impossible to evaluate the effectiveness and safety of these interventions in the treatment of high-risk patients. Large-scale, high-quality RCT studies are needed to evaluate the effectiveness of interventions for HF in dialysis patients. In addition, collaboration between cardiologists and nephrologists is required to design the optimal treatment for these patients due to the influence of HF in ESRD. Dialysis technology is constantly evolving, and a large number of HF patients rely on current dialysis technology to survive, but there are still many problems. Implantable “artificial kidneys” and kidney transplants have made some progress, but there are still many problems to be solved before they can be used in clinical practice. As HF patients on dialysis are often frail, have multiple underlying diseases, and a poor prognosis, future research initiatives should focus more on improving patient quality of life, reducing symptoms, and increasing the number of contingency plans to deal with the poor prognosis of these high-risk patients.

## 11. Conclusions

Dialysis-combined HF populations suffer from numerous complications such as volume overload, potassium abnormalities, renal anemia, calcium and phosphorus metabolism disorders, micro-inflammation and oxidative stress, fluctuations in blood pressure and body weight, and increased lipids, and the management of dialysis-combined HF populations also remains highly controversial. There are also many restrictions on drug use, such as dose reduction or discontinuation, and there is a lack of authoritative clinical studies on drug use, management of dialysis modalities, heart and kidney transplantation, LVAD, and CRT in dialysis-combined HF populations. Patient expectations, comorbidities, age, and quality of life must be taken into account when considering dialysis modality optimization and the choice of which dialysis modality is more beneficial for dialysis patients. Joint studies in cardiac and renal disciplines are still needed to develop rational treatment strategies for dialysis-combined HF populations.
